# Serum PTH reference values in an adult Brazilian population: implications for the diagnosis of hyperparathyroidism

**DOI:** 10.20945/2359-3997000000117

**Published:** 2019-03-18

**Authors:** Pedro Weslley Rosario, Maria Regina Calsolari

**Affiliations:** 1 Santa Casa de Misericórdia de Belo Horizonte Santa Casa de Belo Horizonte Belo Horizonte MG Brasil Santa Casa de Belo Horizonte, Belo Horizonte, MG, Brasil

**Keywords:** Normal PTH, primary hyperparathyroidism, normocalcemic, secondary hyperparathyroidism, diagnosis

## Abstract

**Objective:**

To define serum parathyroid hormone (PTH) reference values in carefully selected subjects following the recommended pre-analytical guidelines.

**Subjects and methods:**

First, 676 adults who would be submitted to thyroidectomy were evaluated. Patients using interfering medications or with malabsorption syndrome, hypomagnesemia, hyper- or hypophosphatemia, hypo- or hypercalcemia, 25-hydroxyvitamin D < 30 ng/dL, estimated glomerular filtration rate < 60 mL/min/1.73 m^2^, urinary calcium/creatinine ratio ≥ 0.25, thyroid dysfunction, parathyroid adenoma detected during surgery were excluded. The sample consisted of 312 subjects.

**Results:**

The median, minimum, maximum, and 2.5^th^ and 97.5^th^ percentiles of the PTH values obtained were 30, 7.2, 78, 10.1, and 52 pg/mL, respectively. Thus, the reference range was 10 to 52 pg/mL. PTH > 65 pg/mL, the upper limit of normal according to the manufacturer of the kit, was observed in only one subject (0.3%). Considering the upper limit proposed by the kit's manufacturer, 1/6 hypercalcemic patients and 4/8 normocalcemic patients with PHPT had normal PTH. Using the upper limit established in this study, only one normocalcemic patient had normal PTH. Thus, the sensitivity of PTH in detecting asymptomatic primary hyperparathyroidism (PHPT) using the values recommended by the kit and established in this study was 64% and 93%, respectively (50% versus 87.5% for normocalcemic PHPT).

**Conclusion:**

The upper reference limit of PTH obtained for a rigorously selected sample was 20% lower than that provided by the assay, which increased its sensitivity in detecting PHPT.

## INTRODUCTION

The measurement of parathyroid hormone (PTH) is necessary for the diagnosis of hyperparathyroidism. In the presence of hypercalcemia, reduced PTH is expected and normal concentrations are sufficient for the diagnosis of primary hyperparathyroidism (PHPT) (1). In this situation, overestimated limits of normal PTH are unlikely to compromise the diagnosis. A more serious consequence exists in normocalcemic subjects in whom an overestimated upper limit of PTH may mask the diagnosis of normocalcemic PHPT or secondary hyperparathyroidism (SHPT).

The minimum requirements for the definition of PTH reference values is that (i) a reasonable number of apparently healthy subjects are evaluated, that these subjects are (ii) normocalcemic, (iii) have vitamin D sufficiency, (iv) do not have moderate or severe chronic kidney disease, and (v) do not use medications known to interfere with the concentrations of this hormone. A recent review shows that few studies have met all of these criteria so far (2). Even among these few studies, many defined vitamin D sufficiency as concentrations > 20 ng/dL (2). Although this definition is widely accepted, specifically to establish normal PTH values, the cut-off value of 30 ng/dL seems to be more adequate since PTH elevation can occur at vitamin D concentrations between 20 and 30 ng/dL (2). Furthermore, other factors that can elevate PTH in apparently healthy subjects, both recognized as a cause of SHPT and included in the differential diagnosis of normocalcemic PHPT such as hypercalciuria and conditions of malabsorption (1), were not excluded in the previous studies. In addition to the selection criteria of the subjects, guidelines on blood collection (time and need for fasting) and processing must be followed for the measurement of PTH (2).

As we have seen, studies establishing serum PTH reference values in carefully selected subjects following the recommended pre-analytical guidelines remain desired goals. This was the objective of the present prospective study which used an assay commonly employed for the measurement of PTH.

## SUBJECTS AND METHODS

### Study design

The study was prospective and was approved by the local Research Ethics Committee (3).

### Patients

First, 676 adults (age ≥ 18 years) with nodular thyroid disease who would be submitted to bilateral thyroidectomy (3) were evaluated. The subjects were submitted to the measurement of calcium (total and ionized), magnesium, phosphorus, 25-hydroxyvitamin D, creatinine, TSH, PTH, and urinary calcium and creatinine. The following patients were excluded: patients using diuretics, lithium, bisphosphonates, denosumab, recombinant PTH, corticosteroids and calcium or vitamin D supplements, and patients with primary aldosteronism [investigated in the recommended situations (4)], known malabsorption syndrome, hypomagnesemia, hyper- or hypophosphatemia, hypo- or hypercalcemia, 25-hydroxyvitamin D < 30 ng/dL, estimated glomerular filtration rate (eGFR) < 60 mL/min/1.73 m^2^, urinary calcium/creatinine ratio ≥ 0.25, or thyroid dysfunction. The remaining patients also underwent measurement of tissue anti-transglutaminase IgA antibodies and those with a positive result were excluded. Using these criteria, 356 participants were excluded.

### Parathyroidectomy

Trained surgeons performed the surgery of all patients included in the study. The four parathyroid glands were fully explored and grossly abnormal parathyroid glands, i.e., enlarged, were removed. Eight patients had parathyroid adenoma detected during surgery (5,6) and were also excluded. Thus, the final sample consisted of 312 subjects (Table 1).

**Table 1:** Characteristics of the subjects studied

Number of patients studied	312
Sex	Female: 232
	Male: 80
Age (years)	18 to 70 (median: 48)
	< 60 years: 242
	>-60 years: 70
Menopause status	Premenopausal: 130
	Postmenopausal: 102
BMI (kg/m^2^)	18 to 33 (median: 25)

BMI: body mass index.

### Methods

Total calcium [corrected for low albumin (< 4 mg/dL) using the formula: (4 - albumin) x 0.8 + Ca] (reference range: 8.4 to 10.4 mg/dL) and urinary calcium were measured by a colorimetric method. Ionized calcium (reference range: 1.12 to 1.32 mmol/l) was measured with a selective electrode and automatic correction for pH variation. Serum PTH was measured with a chemiluminescent assay (Immulite 2000, Diagnostic Products Corporation, Los Angeles, CA), with reference values of 12-65 pg/mL. A chemiluminescence assay was used to measure 25-hydroxyvitamin D. The eGFR was calculated using the Modification of Diet in Renal Disease Study equation. Tissue anti-transglutaminase IgA antibodies were measured by enzyme immunoassay. All measurements were obtained in the morning after fasting for approximately 10 h, and the samples were processed and analyzed immediately after collection.

### Statistical analysis

As reported in previous studies (7–19), the PTH values did not follow a normal distribution. Thus, the normality range was defined as corresponding to 95% of the results and the 2.5^th^ and 97.5^th^ percentiles as corresponding to the lower and upper limit, respectively (7–19). PTH levels were compared between men and women (pre- and postmenopausal) and between subjects < 60 and > 60 years (2) using the Student t-test and Kruskal-Wallis test. The two-tailed Pearson correlation coefficient test was used to analyze the correlation of age and BMI with PTH values.

## RESULTS

The median, minimum, maximum, and 2.5^th^ and 97.5^th^ percentiles of the PTH values obtained were 30, 7.2, 78, 10.1, and 52 pg/mL, respectively. Thus, the reference range was 10 to 52 pg/mL. PTH > 65 pg/mL, the upper limit of normal according to the manufacturer of the kit, was observed in only one subject (0.3%).

No significant difference in PTH values was observed between men and women (pre- and postmenopausal) (Table 2). There was also no statistically significant difference in the results between subjects < 60 versus > 60 years (Table 2), although concentrations were higher in the latter. Finally, no correlation was found between PTH values and age (p = 0.18) or BMI (p = 0.78).

**Table 2:** Comparison of the serum PTH values

	Serum PTH (pg/mL) [range (median)]	p-value
Women (n = 232)	7.2-76 (29.4)	0.9
Men (n = 80)	7.4-78 (30.6)	
Age < 60 years (n = 242)	7.2-65 (27)	0.2
Age >-60 years (n = 70)	8.5-78 (33)	
Premenopausal women (n = 130)	7.2-68 (28.2)	0.3
Postmenopausal women (n = 102)	8-78 (32.8)	
BMI < 25* kg/m^2^ (n = 156)	8.5-78 (31.2)	0.7
BMI > 25* kg/m^2^ (n = 156)	7.8-72 (28.5)	

BMI: body mass index.

* Median.

To analyze the impact of a new reference range of PTH on the diagnosis of asymptomatic PHPT, we reviewed the serum PTH levels of patients diagnosed with this condition among the 676 patients initially evaluated in this study: 6 had hypercalcemic PHPT (3) and 8 had normocalcemic PHPT (5,6). Considering the limit proposed by the manufacturer of the kit, 1/6 hypercalcemic patients and 4/8 normocalcemic patients had normal PTH. Using the upper limit established in this study, only one normocalcemic patient had normal PTH. Thus, the sensitivity of elevated PTH in detecting asymptomatic PHPT using the values recommended by the kit and established in this study was 64% and 93%, respectively (50% versus 87.5% for normocalcemic PHPT).

## DISCUSSION

We will first highlight some characteristics of the study. A reasonable number of subjects were evaluated. Rigorous selection was performed, following not only the traditionally required criteria [normocalcemia (in this study, total and ionized calcium); vitamin D sufficiency (defined as concentrations > 30 ng/dL); absence of chronic kidney disease, and no use of interfering drugs (2)], but also excluding other factors that can alter PTH in apparently healthy individuals (e.g., hypercalciuria, conditions of malabsorption, hyperphosphatemia, hypomagnesemia), which were not considered in previous studies (2,18,19). In addition to the selection criteria of the subjects, for the measurement of PTH, the guidelines on blood collection and processing were followed (2). The assay chosen is commonly used for the measurement of PTH in Brazil. Finally, it is possible that the inadvertent inclusion of subjects with asymptomatic and normocalcemic PHPT overestimates normal PTH concentrations. However, in the present study, all subjects were submitted to surgical exploration of the parathyroid glands, which were apparently normal.

We found an upper limit that was 20% lower than that provided by the manufacturer of the kit (52 pg/mL instead of 65 pg/mL). As previously seen, an overestimated upper limit may mask the diagnosis of SHPT and especially of normocalcemic PHPT. In fact, 3/8 cases of normocalcemic PHPT from our series (5,6) would have their diagnosis masked if the values proposed by the kit and not the upper limit obtained in this study were used.

A significant influence of sex, age or BMI on PTH concentrations was not observed in the present study. Any observed differences in PTH concentrations might be due to differences in vitamin D concentrations, eGFR, comorbidities and medication use, which was not the case in the present study in which the subgroups were uniform in terms of the selection criteria (vitamin D > 30 ng/dL, eGFR > 60 mL/min/1.73m^2^, no comorbidities or use of interfering medications). However, we recognize that any difference may not have been detected because of the size of the subgroups (18).

In conclusion, the upper limit of the serum PTH reference range obtained for a sample of rigorously selected subjects was 20% lower than that provided by the assay, which increased the sensitivity of PTH in detecting normocalcemic PHPT.
